# Supplementary material for: Predictors of in-hospital mortality in stroke patients
Source: PLOS Glob Public Health. 2023 Feb 8;3(2):e0001278. doi: 10.1371/journal.pgph.0001278 (PMC10021536; doi:10.1371/journal.pgph.0001278)
Supplement: Attachment — Submitted filename: Response to Reviewers.docx [file pgph.0001278.s001.docx]

**To the Editorial Board of the PLOS Global Public Health**

**30 November 2022**

**Dear Dr Colloins Otieno Asweto**

**Re: Manuscript ID- PGPH-D-22-01678**

**We are grateful to you and the reviewers for their constructive reports.**

**In the following, we address the issues raised by each reviewer in a point-by-point manner. Modifications in the manuscript are highlighted in red (track changes)**

**Reviewer #1:**

This is a hospital-based study conducted in Sri Lanka that focuses on identifying potential predictors of in-hospital mortality in stroke patients. The study comprises a cohort of 246 patients who were admitted to a facility in the country. Clinically and radiologically confirmed stroke patients were prospectively evaluated throughout their stay at the hospital. After considering various factors, the study identified patients with glasgow coma scale (GCS) score < 10 and hemorrhagic stroke type are at higher disk of mortality compared to others. As this population-based study is one of the first in Sri Lanka, it may serve as a platform for clinical researchers and physicians to further understand the management of stroke patients. The study will further help to conduct similar research in other parts of the country. This study is based on 246 patients, so it is important to conduct similar research on a larger cohort.

As the researchers have stated that specialty stroke centers are unavailable in the region, the patient care approach differs compared to western healthcare facilities. Therefore, this study is limited to the subcontinent, which has similar healthcare settings. Also, the researchers have not published the data about the length of the stay of the patients considered in this study at the hospital. It will be helpful to know how long on an average patient stayed in the hospital after admitting for stroke.
In "Introduction"- in the third line, statistics are included of 2013, it would be more appropriate if recent statistics are presented.

**Response:**

**We thank the reviewer for the comments**

1) The reviewer has suggested to include the average length of the hospital stay after admitting for stroke. We also feel it is essential information and it was included in the results section
2) The reviewer has suggested to include recent statistics in the introduction. Recent statics were included and the reference list was modified accordingly

**Reviewer #2:**

General; Clear and simple language was used
Background; concise and enables the reader to understand what is intended to be done. The authors however did not show whether stroke is a big concern in Sri Lanka
Methods; Detailed and clear
Results; some of the row percentages do not add up to 100
Discussion; repetition of results in the first paragraph

**Response:**

**We thank the reviewer for the comments**

1) Reviewer has pointed out that we have not shown whether stroke is a big concern in Sri Lanka. We also feel it should be included in the introduction. Since Sri Lankan population is also ageing as many other countries in the world, stroke burden is getting more problematic.
2) Row percentages were revised according to reviewer’s suggestion
3) Reviewer has suggested that repetition of results is there in the first paragraph of discussion. We wanted to give a summary of the results at the beginning of the discussion. However as the reviewer suggested we feel repetition of some of the findings is not important. Hence those are removed in the first paragraph of the discussion.

Again, We thank you and the reviewers for the opportunity to revise this manuscript, and look forward to the final decision.

Yours Sincerely

Vindya Shalini Ranasinghe, on behalf of all authors
